# Infected plastic biliary stent identified by FDG PET/CT: A case report

**DOI:** 10.1097/MD.0000000000047420

**Published:** 2026-02-06

**Authors:** Yeon-Hee Han, Seong-Hun Kim, Chang-Seop Lee

**Affiliations:** a Department of Nuclear Medicine, Research Institute of Clinical Medicine of Jeonbuk National University-Biomedical Research Institute of Jeonbuk National University Hospital, Cyclotron Research Center, Molecular Imaging and Therapeutic Medicine Research Center, Jeonbuk National University Medical School and Hospital, Jeonju, Jeonbuk, Republic of Korea; b Department of Internal Medicine, Research Institute of Clinical Medicine of Jeonbuk National University-Biomedical Research Institute of Jeonbuk National University Hospital, Jeonbuk National University Medical School and Hospital, Jeonju, Jeonbuk, Republic of Korea.

**Keywords:** FDG PET/CT, infected stent, recurrent episodes

## Abstract

**Rationale:**

The diagnostic evaluation of patients presenting with fever can be challenging, especially in cases where conventional imaging modalities fail to reveal a definitive focus of infection. This case illustrates the diagnostic value of F-18 fluorodeoxyglucose positron emission tomography/computed tomography (FDG PET/CT) in detecting infected biliary stents as a persistent nidus of sepsis.

**Patient concerns:**

An 82-year-old woman presented with recurrent episodes of fever, chills, and myalgia. Despite multiple hospitalizations and temporary improvement with empirical antibiotics, her symptoms recurred after discharge.

**Diagnoses:**

FDG PET/CT revealed focal FDG uptake at the proximal tip and linear hypermetabolism along the distal portions of the biliary stents, suggestive of infection. Endoscopic examination confirmed major duodenal papillitis, diffuse biofilm formation, and biliary sludges at the stent tips. Microbiological cultures identified *Escherichia coli* and *Klebsiella pneumoniae*.

**Interventions:**

Endoscopic removal of the biliary stents was performed, followed by targeted antimicrobial therapy based on culture results.

**Outcomes:**

The patient experienced resolution of febrile episodes and improvement in laboratory inflammatory markers following stent removal and appropriate antibiotic treatment.

**Lessons:**

FDG PET/CT could serve as a valuable diagnostic modality in patients with recurrent fever and a history of biliary intervention, by enabling the identification of infected indwelling stents. Early use of FDG PET/CT could help avoid unnecessary procedures and guide timely interventions.

## 1. Introduction

Fever is a common and complex clinical condition that often poses a significant diagnostic challenge. Although most cases are eventually attributed to infections, malignancies, or inflammatory diseases, a considerable proportion remains elusive even after extensive evaluation.^[1,2]^ The difficulty is heightened when routine laboratory and imaging findings are nonspecific or inconclusive, leaving clinicians with limited clues to determine the underlying etiology.

In patients with prior surgical or endoscopic procedures, such as biliary interventions, the diagnostic process becomes even more complicated. Occult or low-grade infections may persist after these procedures and can trigger recurrent sepsis. When standard imaging fails to identify a clear focus of infection, these procedure-related factors must be carefully considered as potential contributors to persistent fever.

Endoscopic biliary stenting is a widely accepted and minimally invasive procedure for managing biliary obstruction, including choledocholithiasis and malignant strictures.^[3]^ However, long-term indwelling stents carry specific infection risks. Over time, these stents can become colonized by bacteria, leading to chronic infections that are difficult to eradicate. This process frequently involves the development of biofilms or sludges within or around the stent, further increasing the risk of persistent or recurrent infection.

Conventional imaging modalities, such as abdominal ultrasound and computed tomography (CT), often lack sensitivity in detecting subtle infectious processes associated with biliary stents. These techniques may reveal indirect signs such as biliary dilatation or pneumobilia but often fail to localize the actual infectious focus, especially when there is no accompanying anatomical abnormality. As a result, patients may undergo repeated empirical antibiotic treatments without definitive resolution, thereby increasing the risk of antibiotic resistance and associated morbidity.^[4,5]^

Fluorine-18 fluorodeoxyglucose positron emission tomography/computed tomography (FDG PET/CT) has been widely used in the oncologic field, where false positives related to inflammation have traditionally been considered a major limitation. However, this limitation becomes an advantage in the context of infection, and FDG PET/CT has emerged as a valuable imaging modality in the evaluation of fever of unknown origin.^[6]^ Its ability to detect metabolically active inflammatory or infectious lesions offers a distinct advantage in cases where conventional imaging is nondiagnostic. In particular, FDG PET/CT can identify hypermetabolic foci suggestive of infection in stents or other prosthetic devices.^[7]^ Herein, we present a case of recurrent febrile episodes due to infected biliary stents, which was successfully diagnosed using FDG PET/CT and subsequently managed with stent removal and targeted antibiotic therapy.

## 2. Case presentation

An 82-year-old woman presented to the emergency department with fever, chills, and myalgia. She had a medical history of choledocholithiasis and had undergone endoscopic retrograde biliary drainage with a plastic biliary stent about 3 years ago. She had required hospitalization on 4 occasions over the past 3 months due to recurrent febrile episodes. Although she responded to empirical antibiotic therapy each time, her symptoms recurred after discharge, raising suspicion of a persistent infectious nidus. Blood cultures were obtained during each hospitalization; however, in most instances, including the current admission, empirical antibiotics had already been administered prior to sampling. She denied abdominal pain, and physical examination revealed no focal tenderness. However, laboratory tests revealed an elevated white blood cell count of 13,240/μL (normal range: 4800–10,800) with neutrophilic predominance at 88.0% (normal range: 50–75%), along with elevated inflammatory markers including C-reactive protein at 41.45 mg/L (normal range < 5) and erythrocyte sedimentation rate at 54 mm/hr (normal range < 9). She also had decreased hemoglobin at 7.3 g/dL (normal range: 13.0–16.5) and albumin at 3.3 g/dL (normal range: 3.5–5.2), findings consistent with systemic inflammation.

Although she underwent chest and abdominal radiographs and computed tomography, only the abdominal CT demonstrated mild biliary dilatation and pneumobilia without overt signs of cholangitis or abscess. These findings are nonspecific and can be seen in a wide range of conditions, from normal variations to malignancy, and they often do not indicate a specific culprit lesion. The remaining imaging studies were unremarkable.

**Figure 1.**
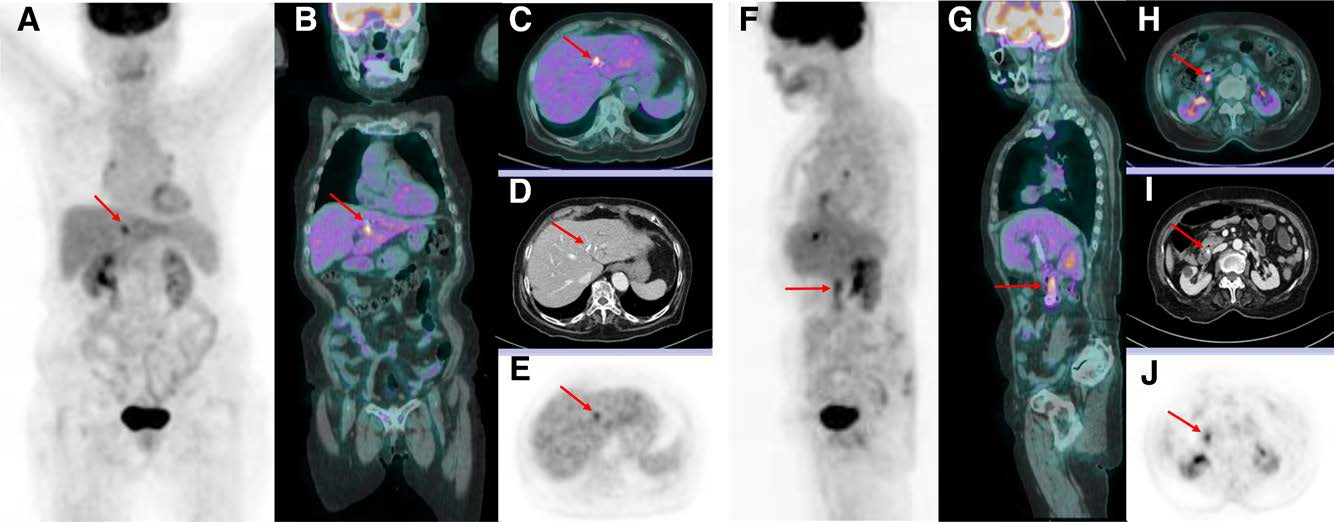
Given her persistent fever of unknown origin, FDG PET/CT was performed to identify occult infection. The scan demonstrated focal FDG uptake at the proximal tip of the stent in the left intrahepatic bile duct (A, coronal maximum intensity projection, B, coronal fusion, C, axial fusion, D, axial CT, and E, axial PET images, red arrows) and linear hypermetabolism along the stents in the distal CBD (F, sagittal maximum intensity projection, G, sagittal fusion, H, axial fusion, I, axial CT, and J, axial PET images, red arrows), suggestive of infected biliary stents. CBD = common bile duct, CT = computed tomography, FDG = fluorodeoxyglucose, PET = positron emission tomography.

**Figure 2.**
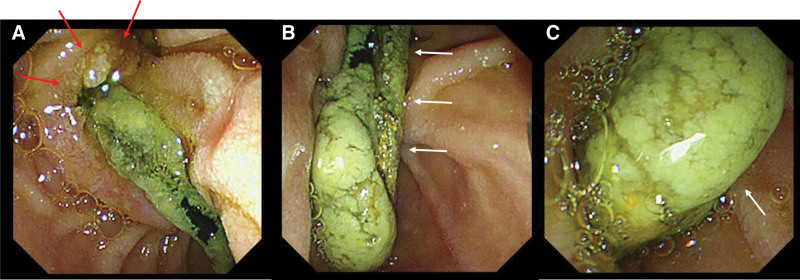
Subsequently, endoscopic removal of the biliary stents was performed, revealing major duodenal papillitis (A, red arrows) and diffuse biofilms along the stents as well as a biliary sludge at the distal tips (B and C, white arrows).

**Figure 3.**
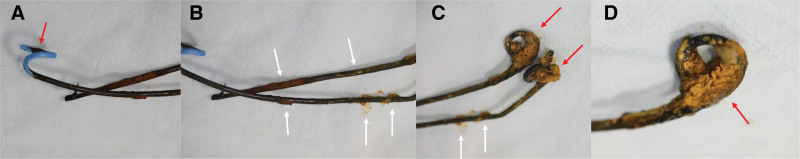
Macroscopic inspection of the stents revealed that the proximal tip of the double pigtail stent was slightly uncoiled and covered with biofilms (A, red arrow), corresponding to the area of focal FDG uptake on PET/CT. Both single and double pigtail stents demonstrated multifocal biofilms particularly the areas located in the distal CBD, ampulla of Vater, and major duodenal papilla, which correlated well with the FDG uptake pattern seen on PET/CT (B and C, white arrows). A biliary sludge was also observed at the distal tips of the stents (C and D, red arrows). CBD = common bile duct, CT = computed tomography, FDG = fluorodeoxyglucose, PET = positron emission tomography.

Given her persistent fever of unknown origin, FDG PET/CT (Fig. 1) was performed to identify occult infection. The scan demonstrated focal FDG uptake (SUV_max_: 5.66) at the proximal tip of the stent in the left intrahepatic bile duct and linear hypermetabolism (SUV_max_: 4.96) along the stents in the distal common bile duct (CBD), suggestive of infected biliary stents. Subsequently, endoscopic removal of the biliary stents was performed, revealing major duodenal papillitis and diffuse biofilms along the stents as well as biliary sludges at the distal tips (Fig. 2). Macroscopic inspection of the stents (Fig. 3) revealed that the proximal tip of the double pigtail stent was slightly uncoiled and covered with biofilms, corresponding to the area of focal FDG uptake on PET/CT. Both single and double pigtail stents demonstrated multifocal biofilms particularly the areas located in the distal CBD, ampulla of Vater, and major duodenal papilla, which correlated well with the FDG uptake pattern seen on PET/CT. Sludges were also observed at the distal tips of the stents. These biofilms and sludges were thought to impede eradication because they protect embedded microorganisms from antibiotics and host immune responses, allowing persistent low-grade infection. At the same time, this ongoing microbial activity leads to focal metabolic activation, which explains the FDG uptake patterns observed on FDG PET/CT.

**Figure 4.**
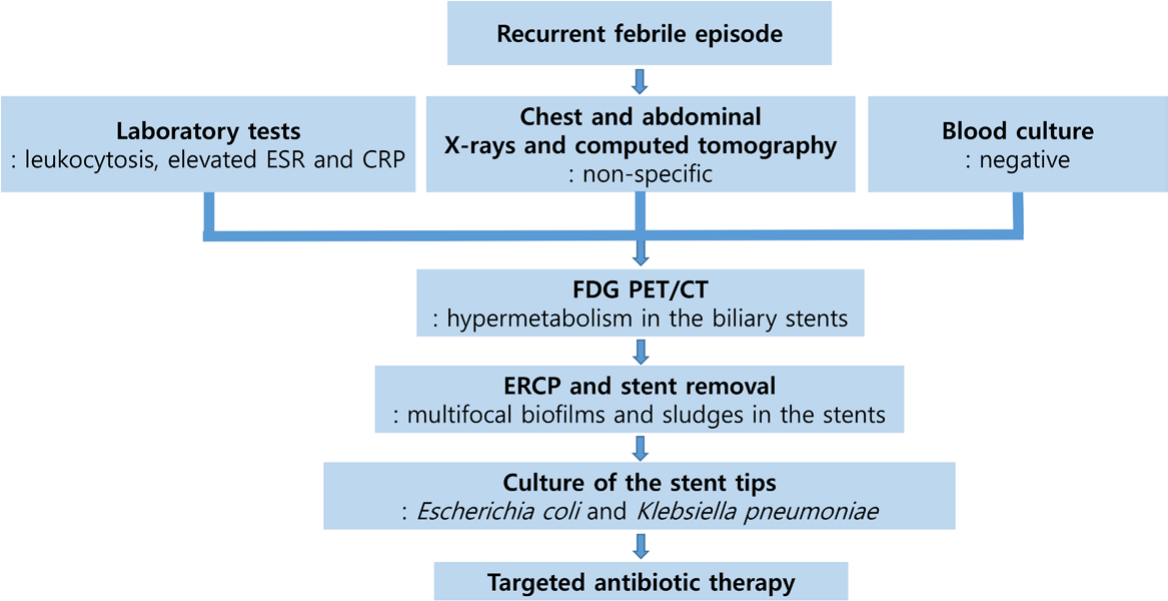
Clinical timeline of the patient. CRP = C-reactive protein, CT = computed tomography, ERCP = endoscopic retrograde cholangiopancreatography, ESR = erythrocyte sedimentation rate, FDG = fluorodeoxyglucose, PET = positron emission tomography.

The combined findings from FDG PET/CT, endoscopic retrograde cholangiopancreatography, and macroscopic inspection of the biliary stents provided strong evidence of infectious colonization of the long-term indwelling stents. Microbiological culture of the stent tip grew *Escherichia coli* and *Klebsiella pneumoniae*, which are known to be the most commonly identified organisms in infected biliary stents. Following stent removal, the patient was treated with intravenous tigecycline (100 mg loading dose, followed by 50 mg every 12 hours) and ceftazidime/avibactam (Zavicefta^®^, 2.5 g every 8 hours) for 14 days, according to the susceptibility profiles of *E coli* and *K pneumoniae*. Both organisms showed multidrug resistance, with *K pneumoniae* producing an OXA-type carbapenemase. The patient responded promptly, with defervescence within 3 days and normalization of inflammatory markers by the end of therapy. She remained afebrile and clinically well during outpatient follow-up. A clinical timeline of the patient is shown in Figure 4.

## 3. Discussion

### 3.1. Complications of biliary stents

Endoscopic biliary stenting is a well-established therapeutic method for patients with biliary obstructive diseases such as bile duct malignancy or benign bile duct stricture.^[8,9]^ Despite its clinical usefulness, it may lead to complications such as stent occlusion, acute cholangitis, proximal or distal migration, and stone formation.^[10]^ Regarding stent occlusion, it is primarily caused by sludge, which consists of microorganisms and various substances, including calcium bilirubinate, dietary fibers, and proteins.^[11]^ A prospective cohort study of biliary plastic stents reported occlusion rates of 11.5% to 13.0% after a median indwelling time of 63 days (range, 1–1274 days) which were associated with a significantly increased risk of cholangitis.^[12]^ The median patency duration of biliary stents has been reported to range from 80 to 126 days.^[13]^ Accordingly, routine stent exchange at intervals not exceeding 3 months is generally recommended to minimize the risk of occlusion. At our institute, we implement a more stringent protocol, limiting the use of the same stent to a maximum of 50 days. In this case, the patient had retained plastic stents inserted at another hospital for approximately 2 years, likely due to noncompliance with follow-up.

### 3.2. Bacterial colonization and biofilm formation

Long-term indwelling biliary stents are prone to bacterial colonization and subsequent biofilm formation, which provides a protective microenvironment that shields bacteria from host immune defenses and antibiotics, thereby facilitating chronic, recurrent infection.^[14]^ The presence of biofilm and biliary sludge on the stent surface, as observed in this case, likely contributed to the recurrent febrile episodes, despite the absence of overt cholangitis or abscess formation on conventional imaging. Although FDG PET/CT cannot visualize biofilm directly, increased metabolic activity around the stent may indirectly reflect inflammation associated with biofilm. These insights highlight the clinical importance of recognizing biofilm biology when evaluating recurrent infections in patients with biliary stents.

### 3.3. Microbiological spectrum and pathogenesis

The most commonly isolated organisms from infected biliary stents include *E coli*, *K pneumoniae*, *Enterococcus faecium*, *Enterococcus faecalis*, and *Pseudomonas aeruginosa*.^[15,16]^ One study reported that pathogens isolated from bile samples included gram-negative organisms (67.05%), Gram-positive ones (29.48%), and fungi (4.56%).^[16]^ In our patient, *E coli* and *K pneumoniae*, gram-negative enteric bacteria frequently implicated in biliary stent infections, were cultured.^[17]^ Reflux of intestinal contents into the bile system, often facilitated by sphincter of Oddi dysfunction, is considered a key mechanism promoting bacterial colonization on the stent surface and subsequent development of an ascending biliary tract infection.^[18]^ Another commonly detected group, gram-positive organisms such as Enterococcus species, has been reported to be more frequently isolated from cholangitis associated with biliary endoprosthesis compared to cases without endoprosthesis.^[19]^

In our case, *E coli* and *K pneumoniae* were both isolated from the removed stent tip, and antimicrobial susceptibility testing revealed multidrug resistance, including resistance to most β-lactams, fluoroquinolones, and trimethoprim/sulfamethoxazole. The *K pneumoniae* isolate exhibited an OXA-type carbapenemase phenotype, which accounts for its carbapenem resistance, while *E coli* remained susceptible to gentamicin and imipenem. These findings are consistent with the growing prevalence of carbapenemase-producing Enterobacteriaceae in device-associated infections, including biliary stents. The coexistence of these organisms within biofilms likely facilitated horizontal gene transfer and enhanced antimicrobial tolerance. Biofilm-embedded bacteria display altered metabolic states and reduced membrane permeability, limiting antibiotic penetration and allowing persistent infection even under broad-spectrum coverage. Such resistance mechanisms highlight the importance of culture-guided therapy and the need to consider combination regimens such as tigecycline or β-lactam/β-lactamase inhibitor combinations when managing infected biliary stents.

In addition, *P aeruginosa*, a gram-negative non-fermenter, is a clinically significant pathogen associated with a high mortality rate in bacteremic biliary tract infections, partly due to the use of inappropriate empirical treatment.^[20,21]^ Culture-directed antibiotic therapy accompanying stent removal is essential for effective management of infected biliary stent. Furthermore, several studies have explored fundamental approaches to preventing biofilm formation, including antimicrobial coatings, antibiotic-eluting stents, and anti-reflux designs.^[22–25]^ To ensure that these appropriate treatments are applied in a timely manner, early detection of infected biliary stents is crucial in clinical practice.

### 3.4. Imaging evaluation and utility of FDG PET/CT

In the present case, CT showed mild biliary dilatation and pneumobilia, findings commonly observed in patients with biliary stents without infection, whereas FDG PET/CT demonstrated significant radiotracer uptake around the biliary stents, corresponding to the sites of biofilm identified during endoscopic inspection. The hypermetabolism observed at the infected biliary stents on FDG PET/CT is thought to result from intense FDG uptake by acute inflammatory cells aggregated in the liver parenchyma around the proximal stent tip and in the distal CBD where the distal portions of the stents were located. This functional imaging modality provides physiologic insights into the inflammatory activity and successfully localizes the infectious focus,^[26,27]^ which in turn prompted earlier consideration of stent removal rather than waiting for overt cholangitis to develop. We believe that the PET/CT findings guided clinicians to proceed with stent removal and to initiate organism-directed therapy.

The findings of this case highlight the need to refine device-related management strategies, particularly regarding the optimal timing of stent exchange and the development of structured monitoring protocols. This approach may reduce reliance on unnecessary empirical therapies and lower the risk of antibiotic resistance. It is particularly useful when infection is clinically suspected but not demonstrable on conventional imaging. Early detection of subtle infectious activity also supports more informed decisions regarding stent exchange and monitoring strategies. Overall, these findings may aid in developing more evidence-based protocols for managing biliary stents in patients prone to recurrent infection.

### 3.5. Future perspective

Further studies are needed to determine how early FDG PET/CT should be incorporated into the diagnostic pathway and to identify the point at which its use yields meaningful clinical benefit. Future work may also investigate optimal surveillance protocols for biliary stents, including appropriate intervals for timely exchange to lower the risk of infection. In addition, the potential advantages of antimicrobial-coated or anti-reflux stents in preventing biofilm formation warrant further evaluation. Finally, examining the cost-effectiveness and real-world accessibility of FDG PET/CT could help clarify its role in routine clinical practice.

### 3.6. Limitations of the case and clinical implications

This case report has several limitations. First, blood cultures showed no bacterial growth, even for the organisms that were later isolated from the stent tips. This was likely due to a masking effect from the empirical antibiotics administered before the blood cultures were taken. Second, FDG PET/CT scans may show increased uptake in noninfectious inflammatory conditions or even physiologic uptake shortly after insertion of stents,^[28]^ which can potentially lead to false-positive interpretations. Larger prospective studies are warranted to validate the clinical utility of FDG PET/CT in the diagnostic evaluation of infected biliary stents. Finally, this case also illustrates the therapeutic challenge posed by multidrug-resistant and carbapenemase-producing organisms in device-related infections, underscoring the necessity of individualized antimicrobial strategies guided by susceptibility testing. Nevertheless, this case report presents FDG PET/CT findings that correlate well with sequential endoscopic retrograde cholangiopancreatography results and the macroscopic appearance of the stents, offering valuable insights into infected biliary stents for clinicians managing such conditions.

In conclusion, FDG PET/CT could serve as a valuable diagnostic modality in patients with recurrent fever and a history of biliary intervention by enabling the identification of infected indwelling stents. Early use of FDG PET/CT could help avoid unnecessary procedures and guide timely interventions. Future studies with larger patient cohorts are warranted to better define its diagnostic performance and to evaluate its potential role in guiding management algorithms.

## Author contributions

**Conceptualization:** Yeon-Hee Han, Chang-Seop Lee.

**Data curation:** Seong-Hun Kim.

**Methodology:** Yeon-Hee Han, Chang-Seop Lee.

**Validation:** Seong-Hun Kim, Chang-Seop Lee.

**Writing – original draft:** Yeon-Hee Han.

**Writing – review & editing:** Seong-Hun Kim, Chang-Seop Lee.
